# Application of the Reverse Line Blot Assay for the Molecular Detection of *Theileria* and *Babesia* sp. in Sheep and Goat Blood Samples from Pakistan

**Published:** 2013

**Authors:** F Iqbal, RM Khattak, S Ozubek, MNK Khattak, A Rasul, M Aktas

**Affiliations:** 1 Zoology Division, Institute of Pure and Applied Biology, Bahauddin Zakariya University Multan 60800, Pakistan; 2 Department of Zoology, Kohat University of Science and Technology, Kohat, Pakistan; 3 Department of Parasitology, Faculty of Veterinary Medicine, University of Firat, 23119 Elazig, Turkey; 4 Department of Zoology, University of Hazara, Mansehra, Pakistan; 5 Changchun Institute of Applied Chemistry, Chinese Academy of Sciences, Changchun 130021, China

**Keywords:** Sheep, Goat, *Theileria; Babesia*, 18S rRNA gene, RLB

## Abstract

**Background:**

The present study was designed to detect the presence of tick-borne parasites (*Theileria* and *Babesia* spp.) in 196 blood samples collected from apparently healthy sheep and goats from two provinces, Punjab and Khyber Pukhtoon Khwa, in Pakistan.

**Methods:**

Reverse line blot (RLB) assay was applied for the parasitic detection by the amplification of hypervariable V4 region of the 18S ribosomal RNA (rRNA) gene. A membrane with covalently linked generic and species specific oligonucleotide probes was used for the hybridization of amplified PCR products.

**Results:**

Parasites were detected in 16% of the ruminant blood samples under study. Two *Theileria* species, *T. lestoquardi* and *T. ovis*, were identified in samples. 25, of the total 32, infected animals were from Khyber Pukhtoon Khwa.

**Conclusion:**

Sheep were more prone to tick borne haemoprotozans as 81% infected samples were sheep as compared to 19% goats (*P* > 0.001). Risk factor analysis revealed that male (*P* = 0.03), animals infested by ticks (*P* = 0.03) and herd composed of sheep only (*P* = 0.001) were more infected by blood parasites.

## Introduction

Livestock has the major contribution in the economy of Khyber Pukhtoon Khwa and Punjab provinces in Pakistan (1). Punjab province contributes 67% buffalo, 46% cattle, 37% goat and 26% sheep in livestock population of Pakistan. Livestock industry is economically more important in KPK as compared to Punjab and Sindh as these provinces have suitable land for crops and well established industrial sectors. According to an estimate in KPK, the livestock contributed 62.8 billion rupees to the national exchequer (2). Ticks are the major vectors of piroplasms (*Theileria* and *Babesia* spp.) and in ruminants tick borne diseases are causing the major economic losses to livestock industry in Asia and more specifically in Pakistan (3) by producing anemia resulting in morbidity and mortality in animals resulting in animal losses, decreased meat, milk and offspring compared to uninfected animal. Several species of piroplasms are transmitted to sheep and goats by Ixodid ticks (4–6).

Classically, piroplasmosis in sheep and goats can be diagnosed by microscopic screening of blood films stained with Giemsa along with the clinical symptoms animals produce in acute cases (7). Occasionally, recovered animals sustain microscopically undetectable sub clinical infections and via potential vectors causing transmission of the disease (8, 9). For the determination of sub clinical infections, serological methods are frequently employed but they lack sensitivity and specificity (10). Therefore, more sensitive and specific DNA amplification methods are used as standard tools for piroplasmosis diagnosis. In recent years, species-specific polymerase chain reaction (PCR) and reverse line blot (RLB) hybridization, which is PCR-based molecular genetics technique have been extensively used for the piroplasm infection in ruminants (11–16).

The present study was designed to use RLB assay for improved and simultaneous detection and identification of multiple *Theileria* and *Babesia* sp. in randomly selected sheep and goat blood samples from various sampling sites in Punjab and KPK in order to develop baselines about the presence of piroplasm in local sheep and goat population and to establish a correlation, if any, between various risk factors, known to be associated with the spread of piroplasmosis, with the presence of piroplasms in small ruminants.

## Materials and Methods

### Data and blood sample collection

Blood samples were collected from 196 clinically healthy small ruminants (82 sheep and 114 goats) from two provinces, Punjab (in and around Multan district) and from Kohat district in Khyber Pukhtoon Khwa, in Pakistan. From 32 randomly selected herds, 10% of apparently healthy animals were blood sampled from jugular vein and preserved by adding 400 µl of 0.5 M EDTA in 5 ml Eppendorf tubes. In order to calculate the risk factors involve in the spread of piroplasmosis, data describing the characters of animal (specie, gender, age, prior treatment for piroplasmosis, presence or absence of ticks) and herd (location, size, composition, association of dogs with the herds and presence or absence of ticks on dogs) was collected on the spot by the investigators during sample collection. Ethic committee of Bahauddin Zakariya University Multan (Pakistan) approved all the experimental techniques and animal handling protocols.

For risk factor calculations, animals were categorized on the basis of their age as young (less than 1-year) and adults (more than 1-year-old). On the basis of herd size, they were divided into two categories: small herds composed of 1-15 and large herds with more than 15 animals. Also, on the basis of herd composition, herds were divided into three categories: herds consist of goat or sheep or herds containing both sheep and goats.

### DNA isolation

Protocol described by Shahnawaz et al. (3) was followed for the DNA isolation by inorganic method. DNA quality was assessed by submerged gel electrophoresis and through optical density counts at 260/280 nm.

### 18S rRNA gene amplification and RLB hybridisation

Approximately 360 and 430 bp fragments of the hypervariable V4 region of the 18S rRNA gene of *Theileria* and *Babesia* was amplified by using the forward (5’-GACACAGGGAGGTAGTGACAAG-3’) and reverse (Biotin-5’-GACACAGGGAGGTAGTGACAAG-3’) primers previously described by Georges et al. (17). The PCR reaction mixture thermoprofile used in this study was modified from Altay et al. (14). In order to identify ovine piroplasms, the biotinylated *Theileria/Babesia* spp. specific PCR products were hybridized with thirteen oligonucleotide probes. The probes and their sequences are presented in Table 1. The primers and oligonucleotide probes containing a N-(trifluoroacetamidohexyl-cyanoethyl, N, N-diisopropyl phosphoramidite [TFA])-C6 aminolinker were synthesised by The Midland Certified Reagent Co., Inc., USA. Followin Altay et al. (14), RLB membrane was prepared, hybridized, and stripped. A volume of 25 µl of PCR product was diluted with 2 x SSPE-0.1% SDS to an end volume of 160 µl. 20mM EDTA was used to rinse the membrane and stored at +4 °C for reuse in fresh EDTA solution.

**Table 1:** Sequence of oligonucleotide probes hybridized on the membrane

Probe	Sequence of oligonucleotide (5’-3’)	Reference
Catchall	TAATGGTTAATAGGA(AG)C(AG)GTTG	Gubbels et al.,1999
*Theileria* spp.	TGATGGGAATTTAAACC(CT)CTTCCA	Nagore et al., 2004a
*Theileria* sp. OT1	ATC TTC TTT TTG ATG AGT TGG TGT	Nagore et al., 2004a
*T. ovis*	TTTTGCTCCTTTACGAGTCTTTGC	Nagore et al., 2004a
*Theileria* sp. OT3	ATTTTCTCTTTTTATATGAGTTTT	Nagore et al., 2004a
*T. lestoquardi*	ATTGCTTGTGTCCCTCCG	Schnittger et al., 2004
*Theileria* sp. MK	CATTGTTTCTTCTCATGTC	Altay et al., 2007
*Theileria luwenshuni*	TCGGATGATACTTGTATTATC	Schnittger et al., 2004
*Theileria uilenbergi*	TGCATTTTCCGAGTGTTACT	Schnittger et al., 2004
*Babesia* spp.	CCT(GT)GGTAATGGTTAATAGGAA	Schnittger et al., 2004
*B. ovis*	GCGCGCGGCCTTTGCGTTACT	Nagore et al., 2004a
*B. motasi*	ATTGGAGTATTGCGCTTGCTTTTT	Nagore et al., 2004a
*B. crassa*	TTA TGG CCC GTT GGC TTA T	Schnittger et al., 2004

Positive control DNAs isolated sheep infected with *T. ovis*, *B. ovis*, *Theileria* sp. OT3, *Theileria* sp. MK (GenBank accession numbers. EF092452, EF092454, EF092455, EF092456 respectively), were used in the assay. Dr. Ana Hurtada (Department of Animal Health, Instituto Vasco de Investigacion Desarrollo Agrario Berreaga, Bizkaia, Spain) had kindly donated us *Theileria* sp.OT1 and *B. motasi* positive blood samples. *T. lestoquardi* positive blood sample was contributed by Dr. Jabbar Ahmed (Department of Immunology and Cell biology, Research Center, Borstel, Germany). The DNA provided by TBD-RLB Kit (Isogen Life Science, Maarssen, The Netherland) for *Theileria* and *Babesia* species was also used as a positive control in RLB hybridization in addition to the above mentioned sources. Ovine genomic DNA extracted from uninfected sheep blood and distilled water was used as negative controls.

### Statistical analysis

Statistical package, Mini Tab (Version 16), was used for data analysis. Fisher's exact test (for 2 x 2 tables) was used to study the association between the presence of piroplasms and various parameters describing the characters of animal and herds.

## Results

**Fig. 1:**
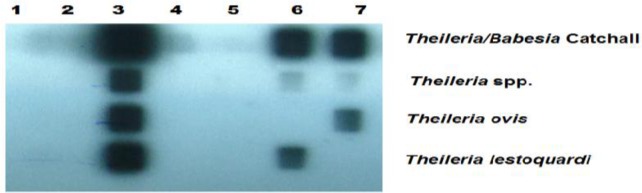
Reverse line blot assay of the PCR products generated by amplification of genomic DNA from sheep samples infected with *Theileria* species. Oligonucleotide probes are shown in rows, and samples are applied in columns. Samples bearing identified single and mixed infections are showed as follows: lane 1, negative control (genomic DNA of uninfected sheep); lane 2, negative PCR control (distilled water); lane 3, mixed infections (*T. lestoquardi*+*T. ovis*); lane 4-5, negative field samples; lane 6, *T. lestoquardi* (single infection); lane 7, *T. ovis* (single infection)

Totally, 32 out of 196 (16%) examined small ruminant blood samples, collected from Southern Punjab and Khyber Pukhtoon Khwa were found piroplasm positive by RLB assay (Fig. 1; Table 2).

**Table 2:** Sampling sites along with the total number of blood samples collected (N) from Punjab and Khyber Pukhtoon Khwa Provinces.% animals infected with parasite and uninfected are given in parenthesis. Fisher's exact test revealed a highly significant (*P* = 0.000) correlation between type of small ruminant (sheep or goat) and incidence of piroplasms

District	n	Piroplasm present n (%)	Piroplasm absent n (%)
Punjab	128	7 (6)	121 (94)
Khyber Pukhtoon Khwa	68	25 (37)	43 (63)
Total Animals (Sheep and Goat)	196	32 (16)	164 (84)
Sheep	82	26 (32)	56 (68)
Goat	114	6 (5)	54 (95)

Prevalence of *Theileria sp*. was significantly different among the two provinces (*P* = 0.000) and incidence of piroplasmosis was higher in KPK as compared to southern Punjab (Table 2) as only 7 out of 128 (6%) blood samples from Southern Punjab were *Theileria* sp. positive as compared to 25 out of 68 (35%) from KPK. Among 32 *Theileria sp*. blood samples, 14 samples were found positive for *T. lestoquardi*, 12 had *T. ovis* while 7 blood samples, all from Kohat district in KPK, were coinfected with both piroplasm species. *Babesia* genus and all its three explored species (*B. ovis, B. crassa and B. motasi*) were not detected in present study among the analyzed samples (Table 2). The 32 piroplasm positive blood samples included 26 sheep (total samples 81, prevalence 23%) and 6 goat (total samples 114, prevalence 11%) samples indicating that sheep were more prone to tick-borne haemoprotozoan than goats (*P* > 0.001).Out of the 2 provinces, 78% (25/32) of the *Theileia* positive samples were present in small ruminants from Khyber Pukhtoon Khwa as compared to 22% (7/32) in Southern Punjab (Table 2). Risk factor analysis revealed that male (*P* = 0.03) and animals infested by ticks (*P* = 0.03) were more infected by blood parasites (Table 3). Statistical analysis of the characteristics of herds indicated that herd composition has significant effect on presence of piroplasm and herd consist of sheep only were more prone to piroplasmosis as compared to herd composed only of goats or having both sheep and goats (Table 4).

**Table 3:** Association between the presence of piroplasms in goats and sheep and the studied parameters describing animal characteristics. Parasite positive and negative % is mentioned in parenthesis

Animal Type	Parameters		No. of Samples	Piroplasm positive n (%)	Piroplasm negative n (%)	*P-value
Sheep and Goat	Sex	Male	81	19 (23)	62 (77)	0.03
		Female	115	13 (11)	102 (89)	
	Age	> 1 Year	28	4 (14)	24 (86)	1.0
		< 1 Year	168	28 (17)	140 (83)	
	Ticks on animals	Absent	142	18 (13)	124 (87)	0.03
		Present	54	14 (26)	40 (74)	

* Probability of Fisher Exact test is mentioned for each parameter (Significant: *P* < 0.05)

**Table 4:** Association between the presence of piroplasm in goats and sheep blood samples and the studied parameters describing herd characteristics. Parasite positive and negative % is mentioned in parenthesis

Parameter		n	Piroplasm present n (%)	Piroplasm absent n (%)	*P-value
Size of Herd	1-15	127	21 (17)	106 (83)	1.0
	15-30	69	11 (16)	58 (84)	
Herd Composition	Goat only	56	4 (7)	52 (93)	0.00
	Sheep only	26	11 (42)	15 (58)	
	Sheep and goat	144	17 (12)	127 (88)	
Association of dog with the Herd	Dog absent	104	15 (14)	89 (86)	0.56
	Dog present	92	17 (18)	75 (82)	
Tick present/absent on dog	Absent	107	16 (15)	91 (85)	0.69
	Present	89	16 (18)	73 (82)	

* Probability of Fisher Exact test is mention for each parameter except herd composition where ANOVA is applied. (Significant: *P* < 0.05)

## Discussion

Identification of parasite species in animals having mixed infection is difficult while using conventional methods. On the other hand, PCR-based techniques allow rapid, sensitive and specific detection of piroplasms. The RLB assay is a powerful tool and practical assay as it is able to simultaneously identify *Theileria* and *Babesia* species and can even detect parasites with extremely low parasitemia (11, 14, 15, 19). To our knowledge, this is the pioneer study in Pakistan in which spp. and *Babesia* parasites are detected through RLB assay.

Comparison of the results revealed that *T. ovis* and *T. lestoquardi* were the only piroplasm species, which were detected in 16% of the sheep, and goat blood samples collected from two provinces in Pakistan (Table 2). Prevalence of *Theileria* sp. was comparatively higher in Khyber Pukhtoon Khwa province (37%) than Punjab (6%). In a recent study, Durrani et al. (7) has reported 27% prevalence of *T. ovis* in small ruminants of Lahore district (Punjab province) which is very high as compared to our overall findings indicating that geographical distribution of land and climatic conditions affects the parasite prevalence. A similar study, conducted by Altay et al. (14), from eastern Turkey, has reported very high prevalence (54%) of piroplasms (*Babesia* and *Theileria* sp.) in sheep while Li et al. (5) has reported even higher prevalence (78%) of *T. ovis* in Xinjiang province of China. In the present study, incidence of piroplasms was very high in sheep (32%) when compared to goat (5%) (Table 2) confirming the findings of Altay et al. (14) and Durrani et al. (20) that sheep are more prone to blood parasites.

We observed that the animals having ticks present (26%) on them had higher incidence of ovine piroplasmosis indicating a positive correlation between the vector ticks and incidence of the disease (Table 3). These observations confirm the previous findings of Durrani et al. (20) and Aktas et al. (13) that ticks are the vectors for the transmission of blood parasites in ruminants.

Herds composed of sheep had higher parasitic prevalence when compared with goat herds or herds having both sheep and goats indicating that composition of herd is also associated with the ovine piroplasmosis (Table 4). Various tick species are known to be involved in the transmission of *Theileria* sp., and many of them are host specific, so we assume that sheep were more infested with specific vector ticks as compared to goats that may be the reason of higher *Theileria* sp. prevalence in them (4–6). Our finding that sheep has higher prevalence of *Theileria* sp. than goats is in agreement with the results reported by Durrani et al. (20).

All the small ruminant blood samples analyzed during present study were locally rose indicating that the piroplasmosis is endemic in Pakistan. Poverty, especially in small towns and villages, and poor hygienic conditions are the contributing factor in piroplasmosis spread in this region. Veterinarians are not available for the help and guidance of livestock owners in many cases worsening the situation further. The overall conditions can be improved by awarding the common people and livestock owners about the risk factors and preventive measures against ovine piroplasmosis resulting in better animal health and hence improving the income of the owners.

## Conclusion

Sheep were more prone to tick borne haemoprotozans as 81% infected samples were sheep as compared to 19% goats (*P* > 0.001). Risk factor analysis revealed that male (*P* = 0.03), animals infested by ticks (*P* = 0.03) and herd composed of sheep only (*P* = 0.001) were more infected by blood parasites.
